# Sucralose inhibited cell survival through the activation of ER stress in human endothelial progenitor cells

**DOI:** 10.1371/journal.pone.0347149

**Published:** 2026-04-17

**Authors:** Chia-Ying Li, Hung-Yu Lin, En-Pei Isabel Chiang, Hung-Chang Hung, Feng-Yao Tang

**Affiliations:** 1 Department of Surgery, Show Chwan Memorial Hospital, Changhua, Taiwan; 2 Doctoral Program in Tissue Engineering and Regenerative Medicine, National Chung Hsing University, Taichung, Taiwan; 3 Research Assistant Center, Show Chwan Memorial Hospital, Changhua, Taiwan; 4 Department of Post-Baccalaureate Medicine, College of Medicine, National Chung Hsing University, Taichung, Taiwan; 5 Department of Food Science and Biotechnology, National Chung Hsing University, Taichung, Taiwan; 6 Department of Internal Medicine, Nantou Hospital, Ministry of Health and Welfare, Nantou City, Taiwan; 7 Department of Health Care Administration, Central Taiwan University of Science and Technology, Taichung, Taiwan; 8 Biomedical Science Laboratory, Department of Nutrition, China Medical University, Taichung, Taiwan; Noorda College of Osteopathic Medicine, UNITED STATES OF AMERICA

## Abstract

Sucralose, a widely utilized non-caloric sweetener, is frequently added to food and beverage products as a sugar substitute aimed at lowering energy consumption and reducing obesity-related health risks. However, epidemiological studies have indicated a possible association between high intake of sucralose and increased prevalence of coronary artery disease (CAD). Prior research has demonstrated that diminished levels of circulating human endothelial progenitor cells (hEPCs) are linked to a higher risk of CAD. Although sucralose is broadly consumed, its direct biological impact on hEPCs has not been comprehensively characterized. In this study, we investigated the cellular effects of sucralose on hEPCs using a variety of *in vitro* techniques, including assays for viability, migration, capillary-like tube formation, lactate dehydrogenase (LDH) release-cytotoxicity assay, and protein expression profiling by Western blotting. Our results revealed that increased concentrations of sucralose significantly impaired hEPCs viability, motility, and neovasculogenic function, accompanied by increased expression of markers associated with apoptosis, inflammasome activation, and pyroptosis. Mechanistic analysis further demonstrated that sucralose strongly activated endoplasmic reticulum (ER) stress/PERK pathways in these cells. Inhibition of ER stress via 4-phenylbutyric acid (4-PBA) substantially attenuated sucralose-induced cell death and reduced the expression of pyroptosis-related proteins and inflammasome markers. Taken together, these findings suggest that sucralose disrupts hEPCs function in part by triggering ER stress, which promotes both apoptotic and pyroptotic cell death programs.

## 1. Introduction

The increasing global prevalence of metabolic disorders, including obesity and type 2 diabetes, has intensified interest in sugar alternatives that can reduce caloric intake without sacrificing sweetness [1]. Non-nutritive sweeteners (NNS) have garnered particular attention due to their minimal caloric content and wide applicability [1]. Among these NNS, sucralose stands out as one of the most extensively utilized synthetic sweeteners across the globe [2]. It exhibits approximately 600-fold greater sweetness than sucrose and demonstrates excellent thermal and chemical stability, making it suitable for incorporation into a variety of processed food and beverage products [3]. Its use has been approved in numerous countries to assist in controlling calorie intake and addressing obesity-related concerns [2,3].

Although sucralose and other NNS were initially regarded as metabolically inert—largely due to their limited systemic absorption and predominant excretion in unchanged form—more recent studies have begun to challenge this assumption [4]. Evidence now indicates that NNS may exert biological activity through interactions with diverse physiological pathways, including those involving gut microbial populations, insulin signaling, oxidative stress, the cardiovascular system, and inflammatory mechanisms [5–7]. These effects raise concerns about their potential to influence long-term metabolic health and vascular integrity [8]. The implications are especially significant for individuals with existing metabolic risk factors, such as diabetes, who also tend to consume higher quantities of these sweeteners [9].

The vascular endothelium is essential for cardiovascular health, functioning in the regulation of blood vessel integrity and new blood vessel formation [10]. Disruption of endothelial function is a key initiating factor in the pathogenesis of atherosclerosis and related cardiovascular disorders [11]. Bone marrow-derived hemangioblasts give rise to hematopoietic and endothelial lineages, including human endothelial progenitor cells (hEPCs), which circulate in the peripheral blood [11]. These progenitor cells have the capacity to differentiate into mature endothelial cells and are actively recruited to sites of vascular injury, where they participate in neovascularization and endothelial repair [12,13]. Owing to their regenerative properties, hEPCs are thought to play a vital role in maintaining vascular homeostasis and facilitating recovery from endothelial injury, thereby potentially reducing the risk of CAD and stroke [14,15]. Moreover, growing research indicates that the abundance of circulating hEPCs reflects vascular health status and may serve as a prognostic marker for cardiovascular events [11]. On the other hand, increased apoptosis or loss of hEPCs may impair their reparative function, contributing to endothelial dysfunction and vascular disease progression [16,17].

Among the diverse cellular pathways implicated in pathological processes, endoplasmic reticulum (ER) stress has emerged as a key regulatory mechanism [18]. The ER is an essential organelle responsible for sensing extracellular stress and ensuring correct protein folding and intracellular equilibrium [19,20]. When these cellular conditions are disturbed, ER stress is initiated, leading to the activation of the unfolded protein response (UPR) [19,20]. One of these responses is mediated by a set of transmembrane sensors, including protein kinase RNA-like ER kinase (PERK), phosphorylated Eukaryotic Initiation Factor 2 alpha (eIF2α), and CCAAT-enhancer-binding protein homologous protein (CHOP), which collectively work to restore ER function by modulating gene expression and translational activity [19].

Sustained or excessive ER stress, however, may trigger programmed cell death, such as apoptosis and pyroptosis [20–22]. Apoptotic death typically involves the activation of caspase-3, and Poly (ADP-ribose) Polymerase (PARP), whereas pyroptosis is characterized by gasdermin D (GSDMD) cleavage, pore formation in the plasma membrane, and secretion of pro-inflammatory cytokines such as interleukin-1β (IL-1β) [23]. This inflammatory form of cell death is frequently initiated through NOD-like receptor protein 3 (NLRP3) inflammasome activation and caspase-1 cleavage, both of which have been linked to the development of cardiovascular conditions, including atherosclerosis, myocardial ischemia, and vascular mineralization [24–26]. Accumulating evidence indicates that upregulated ER stress contributes to the onset and progression of chronic diseases, particularly those affecting the cardiovascular system [27]. Inhibition of ER stress signaling in endothelial cells has been shown to mitigate vascular damage and alleviate atherosclerotic changes [28].

Although sucralose, a commonly used non-nutritive sweetener, was traditionally regarded as metabolically inert, recent studies have suggested that it may interfere with cellular function [8]. A population-based cohort analysis identified a significant correlation between elevated sucralose consumption and increased coronary heart disease risk, reporting an odds ratio (OR) of 1.31 (95% CI: 1.00–1.71; *P* < 0.05) [29]. However, the mechanistic relationship among sucralose exposure, ER stress signaling, apoptosis, and inflammatory cell death, particularly within the context of vascular regeneration, remains inadequately understood. In this study, we explored the impact of sucralose on hEPCs. Our data revealed that sucralose exposure markedly inhibited the cell survival, migration, and neovasculogenesis of hEPCs. Mechanistically, this cytotoxic effect was mediated through the induction of ER stress, apoptosis, inflammasome and pyroptosis, ultimately compromising EPC survival and impairing the neovasculogenesis function.

## 2. Materials and methods

### 2.1. Reagents and antibodies

Primary antibodies targeting Bax, Bcl-2, cleaved caspase-3 (c-caspase3), cleaved PARP (c-PARP), NLRP3, cleaved caspase-1 (c-caspase1), cleaved IL-1β (c-IL-1β), phosphorylated PERK (p-PERK), PERK, e-IF2 α, ATF6, p-IRE1, IRE1, phosphorylated JNK (p-JNK), and cleaved N-terminal gasdermin D (c-N-GSDMD) were obtained from Santa Cruz Biotechnology (San Jose, CA, USA). Antibodies against phosphorylated eIF2α and lamin A were procured from Cell Signaling Technology (Danvers, MA, USA), while the CHOP antibody was purchased from Genetex (Irvine, CA, USA). The actin antibody, MCDB-131 medium, MTT reagent, 4-phenylbutyric acid (4-PBA; ER stress inhibitor), Bay-11–7082 (NF-κB inhibitor), SB203580 (p38 MAPK inhibitor), SP600125 (JNK inhibitor), N-acetylcysteine (NAC), Z-VAD-FMK (pan-caspase inhibitor), and glycyrrhizin (inflammasome inhibitor) were acquired from Sigma-Aldrich (St. Louis, MO, USA). NE-PER nuclear and cytoplasmic extraction reagents were purchased from Pierce Biotechnology (Rockford, IL, USA). The LDH Cytotoxicity Detection Kit was obtained from Promega (Madison, WI, USA). Fetal bovine serum (FBS) was obtained from Thermo Fisher Scientific (Pittsburgh, PA, USA), and the EGM-2 Bullet Kit was provided by Lonza (Allendale, NJ, USA).

### 2.2. Cell culture

hEPCs were seeded on collagen-coated (50 μg/mL) culture dishes and maintained in MCDB-131 medium supplemented with 10% FBS and the EGM-2 growth supplement. Sucralose stock solution (2 M) was prepared in phosphate-buffered saline (PBS). These experiments were performed using independent biological replicates (at least twice) in this study.

### 2.3. Assessment of cell survival

Detection of cell survival was performed using the trypan blue exclusion assay according to the previously described protocol [30]. hEPCs were seeded at a density of 1 × 10⁵ cells/well and grown in a 24-well plate until treatment with sucralose. These hEPCs were cultured in media containing sucralose (at concentrations of 0.02, 0.1, 0.2, 1, 2 mM) in the presence or absence of 2 mM mannitol (MTL; a negative control) or 5 µM staurosporine (STS; a positive control). The cell survival assay was performed in triplicate and repeated at least twice. After 24 h, the culture media were removed from each plate. hEPCs were detached from each well using a trypsin/EDTA solution and subsequently stained with trypan blue solution. The number of live (unstained) and dead (blue) cells was counted using a hemocytometer under a microscope. Cell viability was expressed as a percentage relative to the control subgroup.

### 2.4. Cell migration assay

The migratory capacity of hEPCs was evaluated using Transwell Boyden chambers equipped with polyvinylpyrrolidone-free polycarbonate filters (8-μm pore size), following the manufacturer’s instructions. Before the assay, hEPCs were labeled with Calcein AM (a green fluorescent dye) and treated with sucralose (0.02, 0.1, 0.2, 1, 2mM) in the presence or absence of 2 mM MTL or 5 µM STS. These labeled cells (at a density of 5 x 10^3^ cells/well) were then seeded into the upper chambers in serum-free MCDB-131 medium. The lower chambers were filled with complete MCDB-131 medium supplemented with 10% FBS to serve as a chemoattractant. Following a 24 h incubation, non-migratory cells on the upper surface of the filter were thoroughly removed using a cotton swab. hEPCs that had migrated to the lower side of the membrane were observed using an Olympus IX-71 inverted phase-contrast microscope. Representative photomicrographs were captured and analyzed using an Olympus DP-71 digital camera and imaging system (Tokyo, Japan). Migration activity, or the migration index, was determined by quantifying the number of fluorescent cells in 10 randomly selected fields per filter.

### 2.5. Tube formation assay

To assess angiogenic capacity, 50 μL of Matrigel (at a concentration of 4 mg/mL) was added to each well of a 96-well plate and polymerized at 37°C. hEPCs (1 × 10⁴ cells/well) were treated with sucralose (0.02, 0.1, 0.2, 1, 2 mM) in the presence or absence of 2 mM MTL or 5 µM STS, seeded on Matrigel and incubated for 24 hours. Tube-like structures were imaged using an inverted phase-contrast microscope, and morphometric analysis was performed using the Olympus DP-71 digital camera and imaging system (Tokyo, Japan).

### 2.6. Annexin V/propidium iodide apoptosis assay

To detect apoptosis, hEPCs were exposed to sucralose (0.02–2 mM) for 24 hours. After removing non-adherent dead cells, 5 × 10⁵ viable cells were stained with Annexin V-FITC and PI in binding buffer. Samples were incubated in the dark at room temperature for 5 minutes and analyzed using BD FACS Canto flow cytometry (BD Biosciences, Franklin Lakes, NJ, USA). Apoptotic populations were quantified based on dual-channel fluorescence detection. Apoptotic stages were defined by quadrant analysis. The Annexin V^┼^/PI^―^ population represented early apoptosis, whereas the Annexin V^┼^/PI^┼^ population represented late apoptosis.

### 2.7. Protein extraction and Western Blot analysis

Cytoplasmic and nuclear protein fractions were isolated using the NE-PER kit containing protease and phosphatase inhibitors. Lysates were centrifuged at 12,000 × g for 10 minutes, and the supernatant was collected for further analysis.

Equal amounts of protein (50 μg) were separated via 10% SDS-PAGE and transferred to PVDF membranes. Membranes were probed with primary antibodies against target proteins including Bax, Bcl-2, c-caspase3, NLRP3, c-caspase1, c-IL-1β, c-N-GSDMD, p-PERK, PERK, p-JNK, p-eIF2α, eIF2α, c-ATF6, p-IRE1 and IRE1. For nuclear proteins such as c-PARP and CHOP, lamin A served as the nuclear loading control. β-actin was used as the cytoplasmic loading control for non-phosphorylated proteins. PERK, eIF2α, and IRE1 proteins were used as the cytoplasmic loading controls for p-PERK, p-eIF2 α, and p-IRE1 proteins, respectively.

### 2.8. LDH cytotoxicity assay

hEPCs (1 × 10⁴ cells/well) were cultured in gelatin-coated 96-well plates and treated with (0.02–2 mM) sucralose in the presence or absence of different pathway inhibitors for 24 hours. These inhibitors include 10 μM BAY-117082(BAY; an inhibitor of NF-kB), 10 μM SB203580 (SB; an inhibitor of MAPK/p38), 10 μM SP600125 (SP; an inhibitor of MAPK/JNK), 5mM 4-PBA (an inhibitor of ER stress), 2.5 mM N-acetyl cysteine (NAC; an inhibitor of oxidative stress), 20 μM Z-VAD-FMK (VAD; an inhibitor of apoptosis), and 100 μM Glycyrrhizin (GA; an inhibitor of inflammasome). Cytotoxicity was quantified by measuring LDH release according to the manufacturer’s protocol.

### 2.9. Statistical analysis

All statistical analyses were conducted using SYSTAT software (Chicago, IL, USA). Differences between control and treatment subgroups were assessed using the Student’s *t*-test, with significance defined at *P* < 0.05. Outcomes assessed included survival indices, migration levels, neovasculogenesis indices, apoptotic levels, cytotoxicity levels, and relative protein expression levels.

## 3. Results

### 3.1. Sucralose significantly suppressed cell survival, migration, and neovasculogenesis of hEPCs

**Fig 1:**
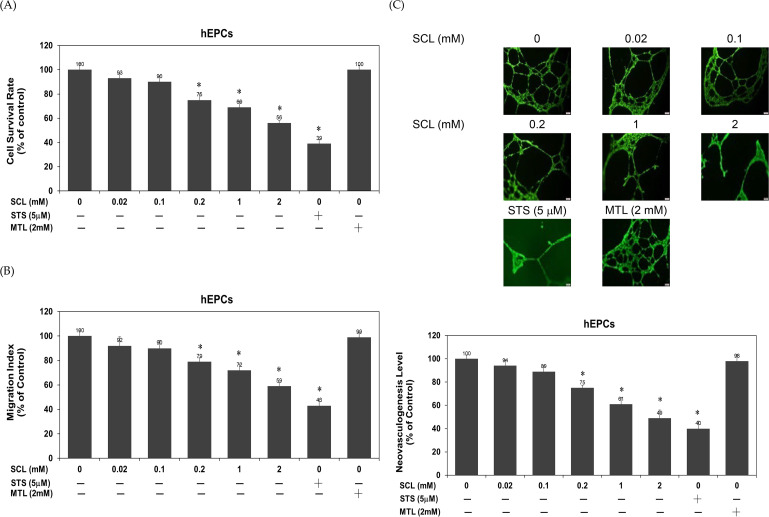
Sucralose significantly suppressed cell survival, migration, and neovasculogenesis of hEPCs. hEPCs were incubated with different concentrations of sucralose (0, 0.02, 0.1, 0.2, 1, and 2 mM) in the presence or absence of 2 mM mannitol (MTL; a negative control) or 5 µM staurosporine (STS; a positive control). These experiments were performed using independent biological replicates (at least twice) in this study. (A) Cell viability was quantified at the 24 h time point using the trypan blue exclusion assay, as described in the Materials and Methods. (B) To assess migratory capacity, hEPCs were treated with identical concentrations of sucralose, mannitol and staurosporine for 24 h, followed by transwell migration analysis, as described in the Materials and Methods. **(C)** Angiogenic function was evaluated via *in vitro* capillary-like tube formation assay under the same treatment conditions. Data are expressed as mean ± standard deviation (SD) from three separate experiments. A single asterisk (*) indicates a statistically significant difference relative to untreated controls (*P* < 0.05).

In this study, we evaluated the potential impact of sucralose on the survival capacity, migratory level, and angiogenic function of hEPCs. We employed staurosporine (STS; 5 µM) as a positive control to induce apoptosis and mannitol (MTL; 2 mM) as a negative control. Our findings demonstrated that sucralose exposure led to a significant, dose-dependent reduction in hEPC cell survival (*P* < 0.05), as illustrated in Fig 1A. Specifically, treatment with sucralose at concentrations of 0.2, 1, and 2 mM resulted in a progressive decline in cell viability, reducing survival by approximately 25%, 31%, and 44% at 24 hours, respectively. To further explore the influence of sucralose on hEPCs function, we assessed cell migratory activity and *in vitro* neovascularization. As shown in Fig 1B, exposure to 0.2, 1, and 2 mM sucralose significantly attenuated cell migration ability. Moreover, the formation of capillary-like structures was markedly compromised across these concentrations, as illustrated in Fig 1C, reflecting a substantial decline in neovasculogenic activity (*P* < 0.05). As expected, the positive control staurosporine (STS; 5 µM) effectively impaired hEPC function, reducing cell survival, migration, and neovasculogenesis by 61%, 57%, and 60%, respectively (Fig 1A–C). Meanwhile, the negative control mannitol (MTL; 2 mM) did not alter cell viability or function, confirming that the observed effects were not due to osmotic stress. Collectively, these results suggest that treatment of high concentrations of sucralose exerts a negative regulatory effect on cell migration and neovascularization, likely in association with the suppression of cell survival in hEPCs.

### 3.2. Treatment of Sucralose significantly enhanced apoptotic cell death in hEPCs

**Fig 2:**
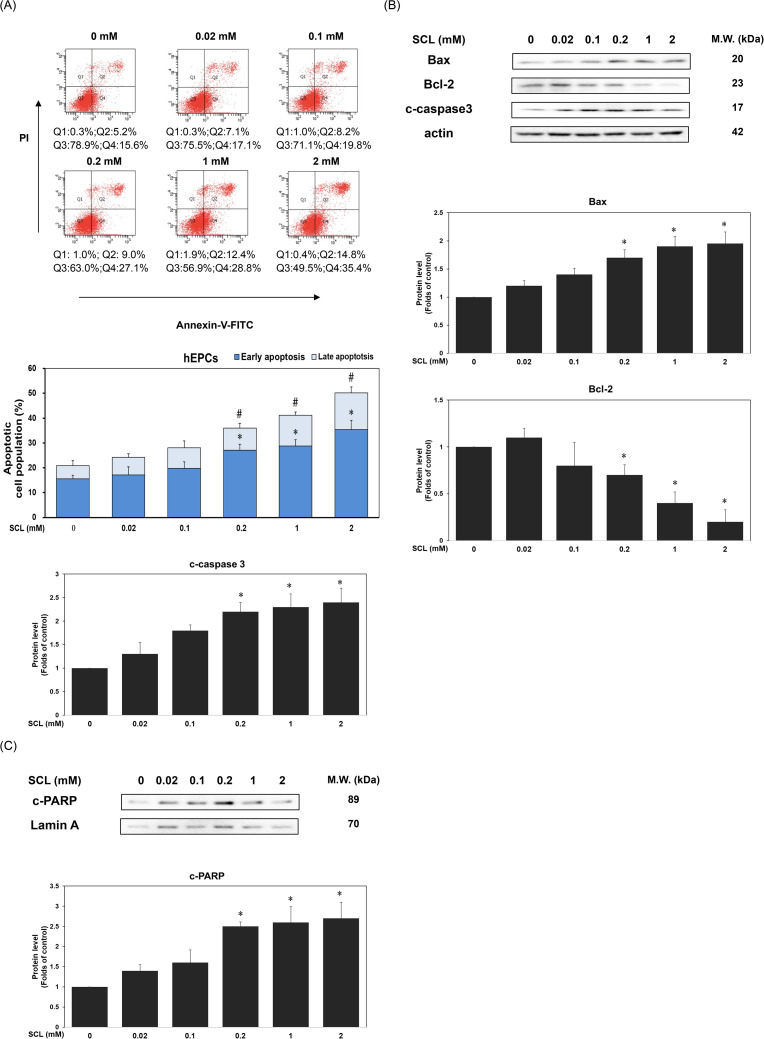
Treatment of Sucralose significantly enhanced apoptotic cell death in hEPCs. hEPCs were treated with varying concentrations of sucralose (0–2 mM) to evaluate apoptotic responses and the expression of apoptosis-related proteins at the 24 h time point. (A) Apoptosis was analyzed by flow cytometry using Annexin V-FITC and propidium iodide (PI) staining following 24 h of exposure. Quantitative data for early and late apoptotic populations are expressed as mean ± SD from three independent experiments. An asterisk (*) denotes a statistically significant difference of early apoptotic population compared to the untreated control group (*P* < 0.05). A pound sign (#) denotes a statistically significant difference of late apoptotic population compared to the untreated control group (*P* < 0.05). (B, C) Western blot analysis was conducted on cytoplasmic and nuclear fractions to determine the expression levels of Bax, Bcl-2, cleaved caspase-3, and cleaved PARP. Densitometric values were normalized to actin and lamin A as internal controls for cytoplasmic and nuclear extracts, respectively. Quantitative protein expression data are presented as mean ± SD from at least two independent assays. Statistical significance relative to the control group is indicated by an asterisk (*, *P* < 0.05).

To investigate the mechanisms by which sucralose affects cell viability, we examined its impact on apoptosis in hEPCs. As depicted in Fig 2A, sucralose exposure led to a statistically significant, concentration-dependent elevation in apoptotic cell populations (*P* < 0.05). Specifically, treatment with 0.2 mM sucralose increased early and late apoptosis to approximately 27.1% and 9.0%, respectively. At 1 mM, the apoptotic fractions rose to 28.8% (early) and 12.4% (late), while 2 mM sucralose further elevated these proportions to 35.4% and 14.8%, respectively, compared to untreated controls (*P* < 0.05). These results suggest that sucralose-mediated survival suppression may be partially attributed to enhanced apoptotic signaling. To delineate the molecular events underlying this response, we assessed the expression of key apoptosis-related proteins. As shown in Fig 2 B and 2C, sucralose treatment upregulated pro-apoptotic markers BAX, cleaved caspase-3 (c-caspase 3), and cleaved PARP (c-PARP), while concurrently suppressing the anti-apoptotic protein Bcl-2 (*P* < 0.05). These findings collectively imply that sucralose impairs hEPCs survival, at least in part, through activation of apoptotic pathways.

### 3.3. Sucralose activated the inflammasome pathway and upregulated key pyroptosis-associated proteins in hEPCs

**Fig 3:**
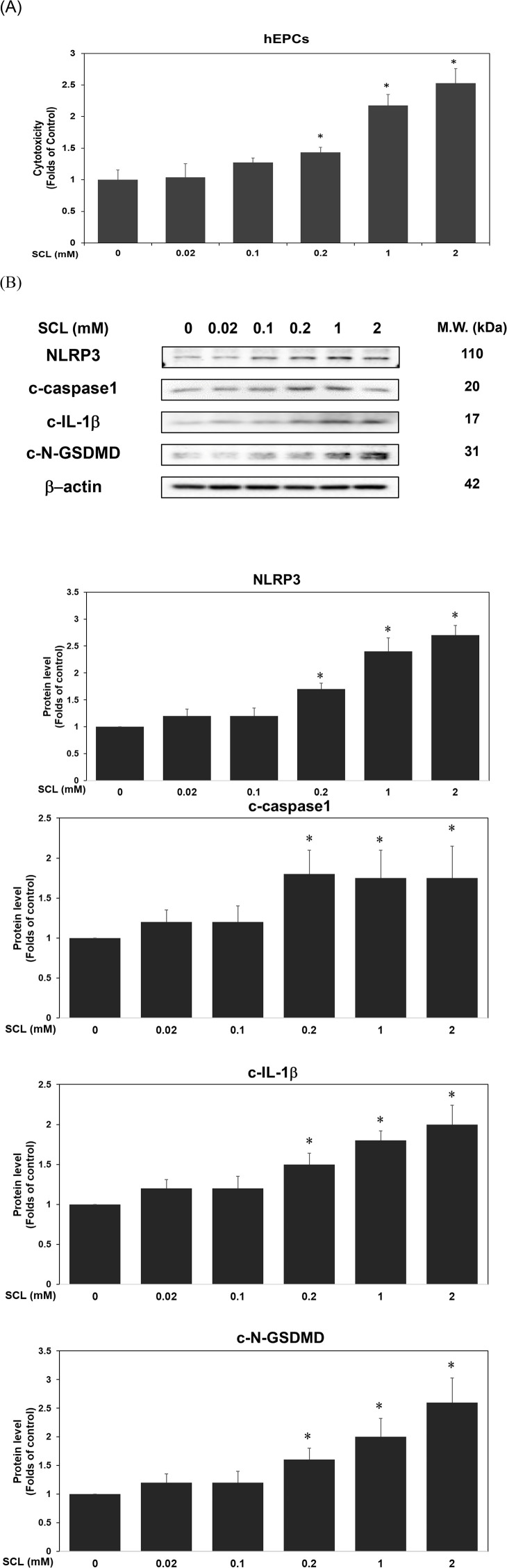
Sucralose activated the inflammasome pathway and upregulated key pyroptosis-associated proteins in hEPCs. hEPCs were treated with varying concentrations of sucralose (0–2 mM) to evaluate cytotoxicity and the expression of inflammasome- and pyroptosis-related proteins at the 24 h time point. Cytotoxicity was analyzed by LDH assay kit following 24 h of exposure according to the manufacturer’s protocol. Quantitative data for cytotoxicity levels are expressed as mean ± SD from three independent experiments. An asterisk (*) denotes a statistically significant difference compared to the untreated control group (*P* < 0.05). Western blot analysis was conducted on cytoplasmic fractions to determine the expression levels of NLRP3, c-caspase1, c-IL-1β, and c-N-GSDMD proteins. Densitometric values were normalized to β-actin as internal controls for cytoplasmic extracts, respectively. Quantitative protein expression data are presented as mean ± SD from at least two independent assays. Statistical significance relative to the control group is indicated by an asterisk (*, *P* < 0.05).

To investigate whether sucralose influences cytotoxicity, we performed a lactate dehydrogenase (LDH) release assay to assess cytotoxicity. As shown in Fig 3A, sucralose exposure led to a significant, dose-dependent increase in LDH release (*P* < 0.05), indicating enhanced membrane damage and cell death. To corroborate these molecular findings, we examined whether sucralose influences inflammasome and pyroptotic signaling in hEPCs. As illustrated in Fig 3B, sucralose treatment resulted in the activation of the inflammasome pathway, accompanied by elevated expression of NLRP3, cleaved caspase-1 (c-caspase-1), and cleaved IL-1β (c-IL-1β). Additionally, we observed a notable increase in the pyroptosis-associated effector cleaved N-terminal gasdermin D (c-N-GSDMD) following sucralose exposure, indicating engagement of the pyroptotic cascade. Together, these data support the conclusion that sucralose not only activates inflammasome signaling but also triggers downstream pyroptotic responses, leading to increased cytotoxicity and potential loss of cell viability in hEPCs.

### 3.4. Treatment of sucralose induced the activation of ER stress in hEPCs

**Fig 4:**
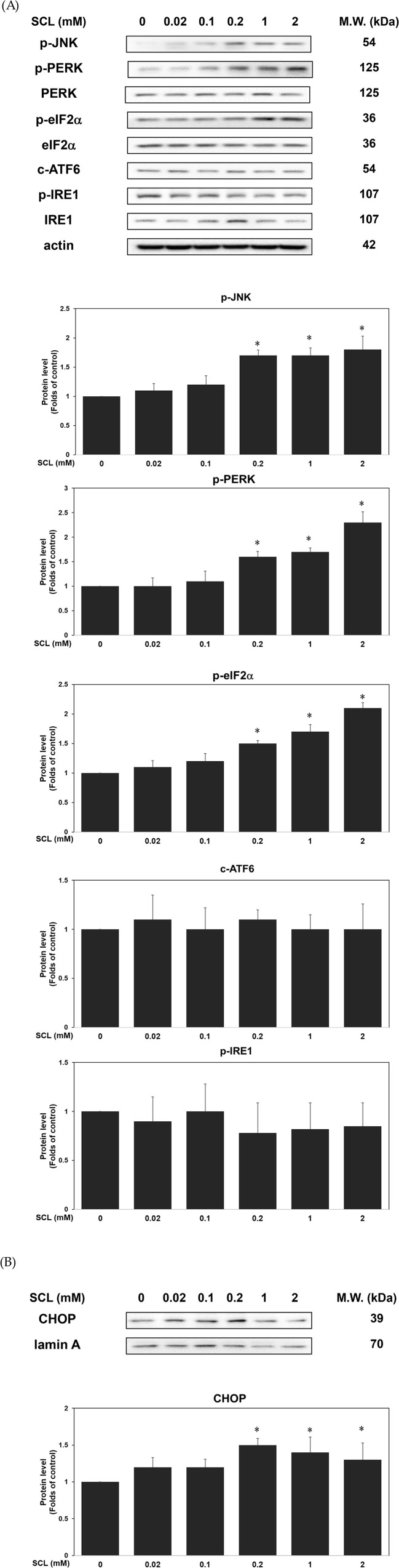
Treatment of sucralose induced the activation of ER stress in hEPCs. hEPCs were treated with varying concentrations of sucralose (0–2 mM) to evaluate the expression of ER stress-related proteins at the 24 h time point. (A-B) Western blot analysis was conducted on cytoplasmic and nuclear fractions to determine the expression levels of p-JNK, p-PERK, PERK, p-eIF2 α, eIF2 α, c-ATF6, p-IRE1 and IRE1 (A) and CHOP (B) proteins. Densitometric values of non-phosphorylated proteins were normalized to α-actin or lamin A as internal controls for cytoplasmic or nuclear extracts, respectively. Densitometric values of p-PERK, p-eIF2 α, and p-IRE1 proteins were normalized to PERK, eIF2 α, and IRE1 proteins, respectively. Quantitative protein expression data are presented as mean ± SD from at least two independent assays. Statistical significance relative to the control group is indicated by an asterisk (*, P < 0.05).

Previous studies showed that ER stress pathways were correlated with programmed cell death, such as apoptosis and pyroptosis [20–22]. To examine the impact of sucralose on ER stress signaling in hEPCs, we examined the expression of key regulatory proteins. As presented in Fig 4A–B, exposure to sucralose resulted in the activation of ER stress-related PERK signaling. Specifically, sucralose treatment led to elevated expression of several ER stress markers, including p-PERK and p-eIF2α proteins. In addition, sucralose promoted nuclear translocation of CHOP, indicating transcriptional activation of these pathways. Sucralose treatment also increased the phosphorylation of JNK signaling protein in hEPCs *in vitro*. However, treatment of sucralose didn’t modulate the expression of ATF6 and the phosphorylation level of p-IRE1 proteins in hEPCs. Collectively, these findings demonstrate that sucralose might stimulate the activation of ER stress/ PERK cascades in hEPCs.

### 3.5. Inhibition of ER stress restored cell survival in sucralose-treated hEPCs

**Fig 5:**
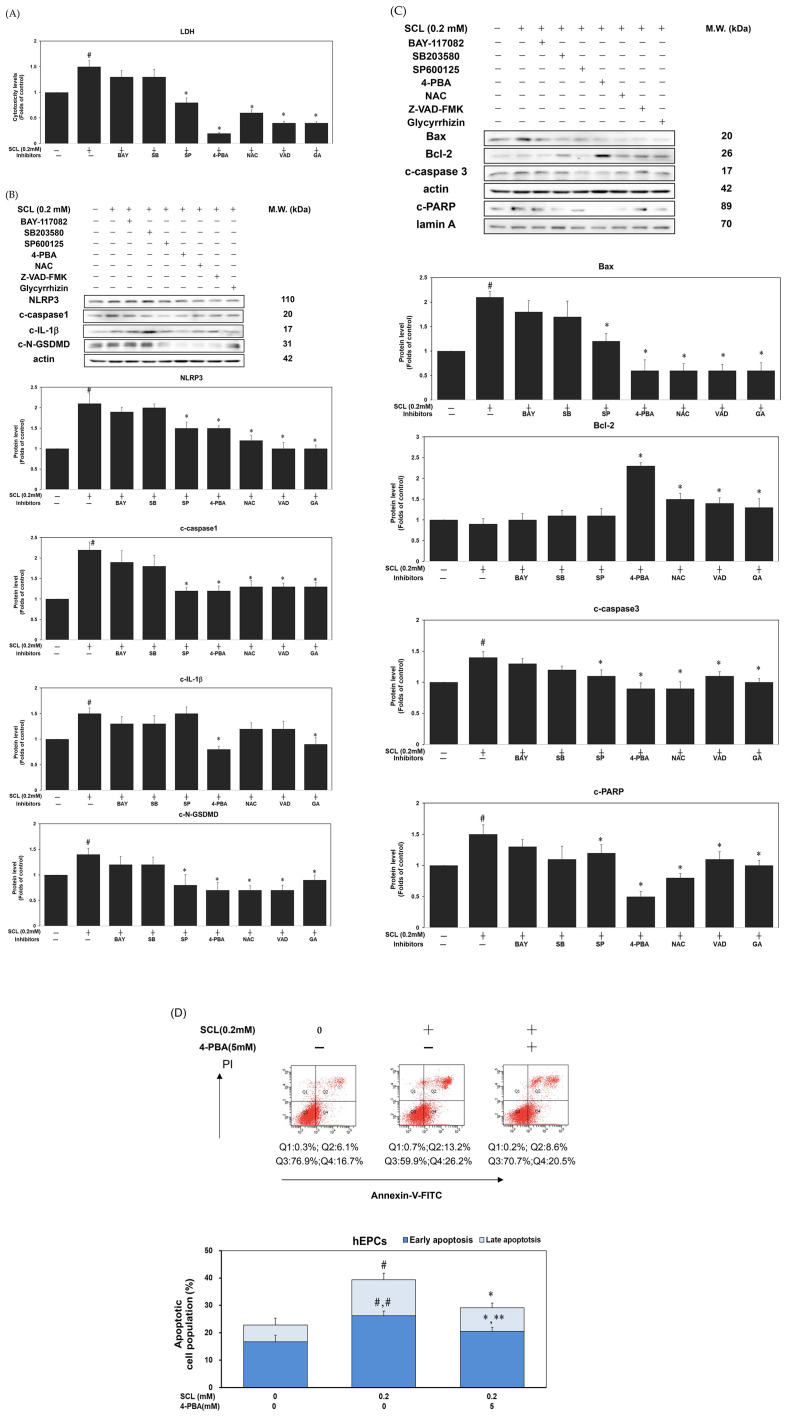
Inhibition of ER stress restored cell survival in sucralose-treated hEPCs. hEPCs were treated with different specific inhibitors in the absence or presence of sucralose (0.2 mM) to evaluate cytotoxicity and the expression of apoptosis-, inflammasome- and pyroptosis-related proteins at the 24 h time point. hEPCs were treated with the following inhibitors, including 10 μM BAY-117082 (BAY; an inhibitor of NF-kB), 10 μ M SB203580 (SB; an inhibitor of MAPK/p38), 10 μ M SP600125 (SP; an inhibitor of MAPK/JNK), 5mM 4-PBA (an inhibitor of ER stress), 2.5 mM N-acetyl cysteine (NAC; an inhibitor of oxidative stress), 20 μM Z-VAD-FMK (VAD; an inhibitor of apoptosis), and 100 μ M Glycyrrhizin (GA; an inhibitor of inflammasome) in the absence or presence of sucralose (0.2 mM) to evaluate cytotoxicity. Cytotoxicity was analyzed by the LDH assay kit following 24 h of exposure according to the manufacturer’s protocol. Quantitative data for cytotoxicity levels are expressed as mean ± SD from three independent experiments. A pound sign (#) denotes a statistically significant difference compared to the untreated control group (*P* < 0.05). An asterisk (*) denotes a statistically significant difference compared to the SCL-treated control group (*P* < 0.05). (B-C) Western blot analysis was conducted on cytoplasmic and nuclear fractions to determine the expression levels of NLRP3, c-caspase1, c-IL-1β, c-N-GSDMD, Bax, Bcl-2, c-caspase3, and c-PARP proteins. Densitometric values were normalized to β-actin or lamin A as internal controls for cytoplasmic or nuclear extracts, respectively. Quantitative protein expression data are presented as mean ± SD from at least two independent assays. A pound sign (#) denotes a statistically significant difference compared to the untreated control group (P < 0.05). An asterisk (*) denotes a statistically significant difference compared to the SCL-treated control group (P < 0.05). hEPCs were treated with sucralose (SCL; 0.2 mM) in the presence or absence of 4-PBA (5mM) to evaluate apoptotic levels. Apoptosis was analyzed by flow cytometry using Annexin V-FITC and propidium iodide (PI) staining following 24 h of exposure. Apoptotic stages were defined by quadrant analysis. The Annexin V^┼^/PI― population represented early apoptosis, whereas the Annexin V^┼^/PI^┼^ population represented late apoptosis. Quantitative data for early and late apoptotic populations were expressed as mean ± SD from three independent experiments. A pound sign (#) denotes a statistically significant difference of late apoptosis level compared to the untreated control group (P < 0.05). A double pound sign (##) denotes a statistically significant difference of early apoptosis level compared to the untreated control group (P < 0.05). An asterisk (*) denotes a statistically significant difference of late apoptosis level compared to the SCL-treated group (P < 0.05). A double asterisk (**) denotes a statistically significant difference of early apoptosis level compared to the SCL-treated group (P < 0.05).

ER stress, oxidative stress, inflammasome and several signaling pathways such as NF-kB and MAPK pathways, could correlate with cell death. To further elucidate the underlying mechanisms involved in sucralose-induced cell death in hEPCs, we adapted a set of selective pharmacological inhibitors against these probable cellular events. As shown in Fig 5A, pretreatment with 4-phenylbutyric acid (4-PBA, an inhibitor of ER stress) significantly mitigated the cytotoxic effects induced by sucralose exposure. Additionally, treatment of Z-VAD-FMK (a pan-caspase inhibitor) and glycyrrhizin (an inflammasome inhibitor) also significantly inhibited cytotoxicity in sucralose-treated hEPCs (*P* < 0.05). Among these inhibitors, 4-PBA exhibited the most pronounced protective effect, suggesting a central role for ER stress in sucralose-mediated cell death. To further elucidate this mechanism, we examined inflammasome and pyroptotic markers following 4-PBA treatment. As shown in Fig 5B, treatment of 4-PBA markedly suppressed the expression of NLRP3, cleaved caspase-1 (c-caspase-1), and cleaved N-GSDMD (c-N-GSDMD). Moreover, 4-PBA markedly suppressed the expression of pro-apoptotic proteins Bax, cleaved caspase3 (c-caspase3), and cleaved PARP (c-PARP) in sucralose-treated hEPCs (Fig 5C). In contrast, levels of the anti-apoptotic protein Bcl-2 were significantly upregulated under the same conditions (Fig 5C). Furthermore, treatment of 4-PBA reversed sucralose-mediated cell apoptosis in hEPCs (Fig 5D). Collectively, these findings demonstrate that sucralose might induce cell death, in part, through stimulating ER stress/ PERK cascades in hEPCs. These findings collectively indicate that ER stress is a key mediator of programmed cell death, activation of inflammasome, and pyroptosis triggered by sucralose in hEPCs.

## 4. Discussion

Excessive consumption of sucralose is correlated with an increased risk of stroke and CAD [29]. A decline number or function of hEPCs is linked to the progression of CAD, particularly atherosclerosis and ischemic vascular injury [31]. Previous studies showed that the risk of ischemic stroke and CAD is inversely correlated with the levels of hEPCs [14]. To better understand the effect of sucralose on vascular functions, the present study investigated cell survival, migration, and neovascularization in hEPCs stimulated with sucralose. Data reveal that sucralose treatment significantly enhanced cell cytotoxicity and cell death (Fig 1 and 3). In addition, sucralose inhibited cell migration and neovascularization activities of hEPCs *in vitro*, as demonstrated by assays of cell migration and tubular formation (Fig 1). The impairment of cell migration and tube formation capacity further supports the notion that sucralose compromises essential regenerative functions in hEPCs. To the best of our knowledge, this is the first report to show the anti-survival, anti-migratory, and anti-neovasculogenic effects of sucralose in the regulation of apoptosis and pyroptosis in hEPCs.

Several lines of evidence support the hypothesis that sucralose may exert its anti-survival activities, in part, through the induction of programmed cell death and pyroptosis. First, we showed that the anti-survival effect of sucralose appeared to be driven by enhanced apoptotic signaling. Flow cytometric analysis revealed a significant elevation in both early and late apoptotic populations following sucralose treatment (Fig 2). Correspondingly, protein expression analyses showed increased levels of pro-apoptotic markers Bax and cleaved caspase-3, alongside downregulation of the anti-apoptotic regulator Bcl-2 (Fig 2B). This molecular signature suggests that sucralose-induced cell death occurs, at least in part, through activation of the intrinsic apoptotic pathway. The induction in c-PARP expression is particularly noteworthy (Fig 2C), as PARP is known to protect cells against extracellular stress by regulating DNA repair. A previous study indicated that the activation of cell apoptosis could lead to dysfunction of EPCs. Indeed, it has been suggested that cell apoptosis may exert its deleterious effects toward dysfunction of EPCs [32].

A second line of evidence that sucralose may exert its deleterious effect on hEPCs through the induction of pyroptosis. In addition to apoptosis, our results revealed that sucralose also activated pyroptotic pathways in hEPCs (Fig 3). Augmentations of cell apoptosis are known as an important mediator of inflammasome activation. Pyroptosis is a pro-inflammatory form of programmed cell death that is increasingly recognized as a contributor to vascular injury and inflammation [33]. A recent study has also demonstrated that activation of apoptosis leads to the activation of the inflammasome and cell pyroptosis [34].

In this study, our results also demonstrated that the sucralose treatment induced the activation of inflammasome and cell pyroptosis (Fig 3B). We observed that sucralose treatment significantly increased the expression of NLRP3 inflammasome components and cleaved caspase-1, accompanied by elevated levels of cleaved N-gasdermin D (c-N-GSDMD), a key executor of pyroptosis. These molecular changes were associated with a dose-dependent increase in LDH release, indicating loss of membrane integrity consistent with pyroptotic cell death.

Mechanistically, to further dissect the upstream signals mediating these death pathways, we evaluated the involvement of ER stress signaling. ER stress is triggered when the protein-folding capacity of the endoplasmic reticulum is overwhelmed, leading to activation of the unfolded protein response (UPR) [19,20]. While initially protective, sustained ER stress can drive both apoptosis and inflammation. Our data demonstrate that sucralose upregulated the expression of phosphorylated JNK protein and multiple ER stress markers, including p-PERK, p-eIF2α, and CHOP (Fig 4). However, treatment of sucralose couldn’t modulate the expression of c-ATF6 (active form of ATF6) and p-IRE1 proteins in hEPCs (Fig 4).

Treatment of Z-VAD-FMK (an apoptosis inhibitor) can suppress the activation of inflammasome signaling proteins such as NLRP3 and c-caspase1 (Fig 5B). Treatment of Z-VAD-FMK further inhibits the activation of cell pyroptosis in association with a decreased expression of c-N-GSDMD pyroptotic biomarker protein (Fig 5B). Additionally, treatment of glycyrrhizin also suppressed the activation of cell pyroptosis in association with a decreased expression of c-N-GSDMD pyroptotic biomarker protein (Fig 5B). These findings align with our hypothesis, suggesting that high dosages of sucralose can inhibit cell survival of hEPCs, indicating a possible association with decreased survival and function of vascular progenitor cells. For the first time to our knowledge, this is the first report showing that sucralose—a widely consumed non-nutritive sweetener—exerts profound inhibitory effects on cell survival of hEPCs, which are essential for vascular repair and neovascularization. Our results demonstrated that sucralose significantly suppressed the survival, migration, and neovasculogenic activity of hEPCs in a dose-dependent manner. These deleterious effects were closely associated with increased apoptotic and pyroptotic cell death, alongside the activation of inflammasome signaling pathways. The dual activation of apoptotic and pyroptotic pathways by sucralose suggests a more complex and synergistic cytotoxic mechanism than previously understood.

Notably, 4-PBA treatment reversed sucralose-induced changes in apoptosis-related proteins in hEPCs. Specifically, 4-PBA reduced Bax, cleaved caspase-3, and cleaved PARP levels, while upregulating the anti-apoptotic protein Bcl-2 (Fig 5C). 4-PBA treatment also reversed sucralose-mediated cell apoptosis in hEPCs (Fig 5D). These results strongly suggest that ER stress is a central mediator of sucralose-induced apoptosis in hEPCs, and that its inhibition can rescue cell survival. Importantly, ER stress plays a pivotal role in priming the apoptotic pathway, which may explain the coordinated induction of pyroptotic signaling observed in our experiments.

These results support the hypothesis that sucralose induces a maladaptive ER stress response, which may serve as a central hub linking cell stress to both NLRP3 inflammasome and pyroptotic outcomes (Fig 5B). Therefore, the activation of ER stress in response to sucralose not only promotes apoptosis but may also amplify downstream inflammasome pathways and pyroptotic cell death in hEPCs. In a brief summary, pharmacological inhibition of ER stress effectively reversed sucralose-induced cytotoxicity and restored cell survival, highlighting the central role of this stress response in mediating the observed cellular dysfunction.

**Fig 6:**
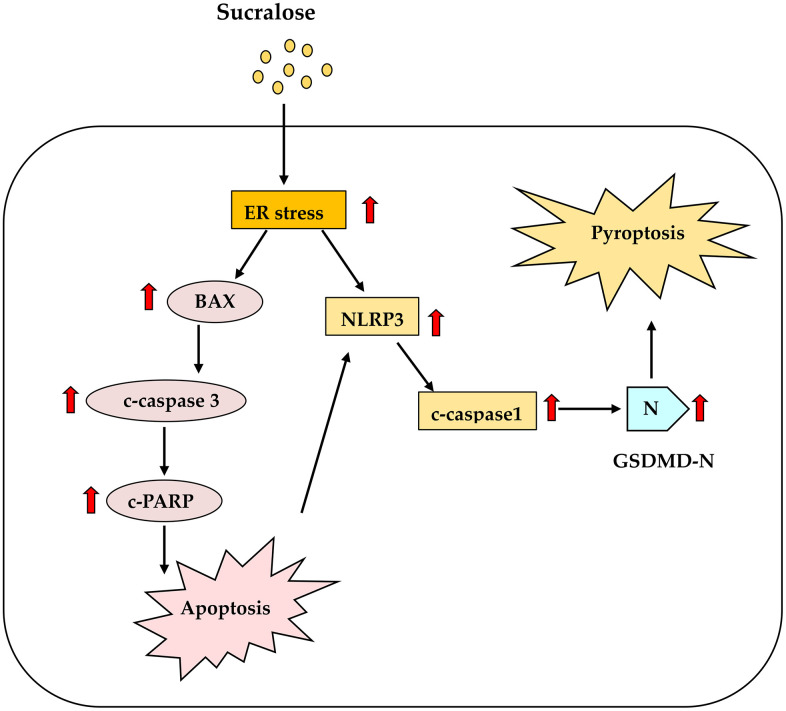
Proposed mechanisms of signaling pathways regulated by sucralose in hEPCs. The proposed mechanism illustrates how sucralose induces critical apoptosis, inflammasome, and pyroptosis pathways, leading to growth inhibition and cell death in hEPCs. ***Red arrows indicate increases in expression level.***
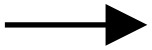
: induction.

Taken together, our findings highlight a novel mechanism by which sucralose, a compound widely regarded as metabolically inert, may compromise vascular repair mechanisms through induction of ER stress/PERK cascades, apoptosis, inflammasome, and pyroptosis in hEPCs. The proposed mechanisms of signaling pathways regulated by sucralose were shown in Fig 6. Previous study demonstrated that consumption of 4 cans of diet sodas will have circulation levels of sucralose 1 ~ 4 μM [35]. The authors selected the sucralose concentrations from 0.2–2 mM to prove the potential hazard. This study was designed to see the maximal possible effect rather than mimicking human eating or drinking patterns. Our study proved the direct effects of high dosages of sucralose on vascular progenitor cells. The clinical relevance of these findings is underscored by recent epidemiological evidence linking sucralose intake with elevated cardiovascular risk [29].

Considering our data, the potential impact of sucralose on hEPCs-mediated endothelial repair warrants further investigation, particularly in populations with pre-existing metabolic or vascular disorders.

## 5. Conclusions

In conclusion, this study provides compelling evidence that sucralose disrupts the function and survival of hEPCs through activation of ER stress and subsequent induction of apoptotic, inflammasome activation, and pyroptotic pathways. These mechanistic insights raise important concerns about the vascular safety of sucralose and suggest that its overconsumption may impair endothelial repair and regeneration, particularly in individuals at high cardiovascular risk. The observed protective effect of ER stress inhibition offers a promising therapeutic avenue to mitigate sucralose-induced hEPCs dysfunction and potentially preserve vascular health. Future studies should examine whether chronic overconsumption of sucralose leads to measurable declines in circulating hEPCs counts or function *in vivo*, and whether these changes correlate with impaired vascular healing or increased cardiovascular events. It would also be valuable to investigate whether other non-nutritive sweeteners elicit similar stress responses in vascular progenitor cells, or if sucralose exerts unique effects.

## Supporting information

S1 Appendix Supporting information for manuscript PONE-D-25-61291R1. (DOCX)
