# Supplementary material for: Sucralose inhibited cell survival through the activation of ER stress in human endothelial progenitor cells
Source: PLoS One. 2026 Apr 17;21(4):e0347149. doi: 10.1371/journal.pone.0347149 (PMC13089890; doi:10.1371/journal.pone.0347149)
Supplement: S1 Appendix — (DOCX) [file pone.0347149.s001.docx]

Supporting Information:

**S2 Fig. Treatment of Sucralose significantly enhanced apoptotic cell death in hEPCs**

| SCL (mM) | 0 0.02 0.1 0.2 1 2 | M.W. (KDa) | |
| --- | --- | --- | --- |
| Bax | 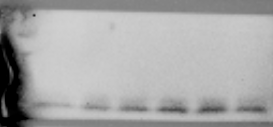 | 20 | |
|  | 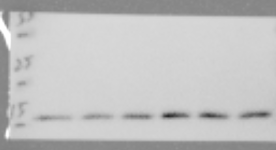 |  | |
| Bcl-2 | 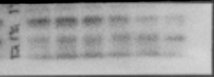 | 23 | |
|  | 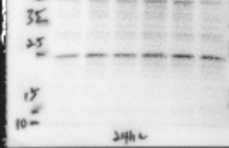 |  |  |
| c-caspase3 | 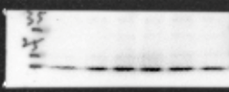 | 17 |  |
|  | 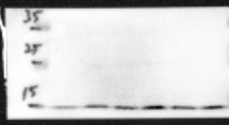 |  |  |
| actin | 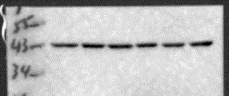 | 42 |  |
|  | 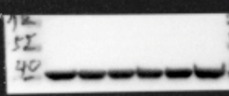 |  |  |
| C-PARP | 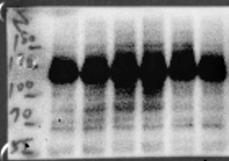 | 89 |  |
|  | 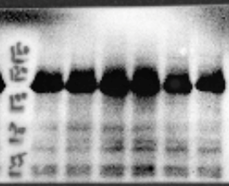 |  |  |
| Lamin A | 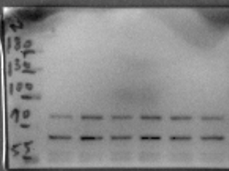 | 70 |  |
|  | 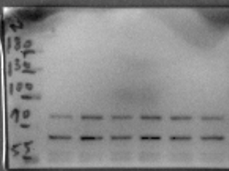 |  |  |

**S3 Fig. Sucralose activated the inflammasome pathway and upregulated key pyroptosis-associated proteins in hEPCs**

| SCL (mM) | 0 0.02 0.1 0.2 1 2 | M.W. (KDa) |
| --- | --- | --- |
| NLRP3 | 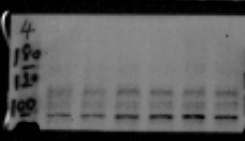 | 110 |
|  | 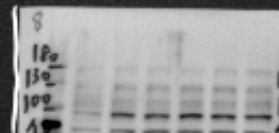 |  |
| c-caspase1 | 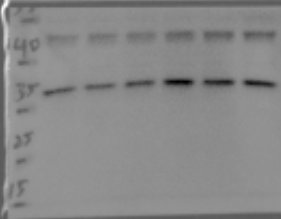 | 35 |
|  | **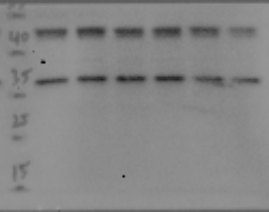** |  |
| c-IL-1β | 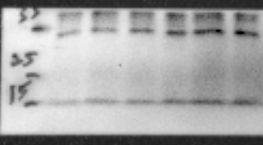 | 17 |
|  | 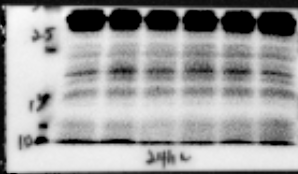 |  |
| c-N-GSDMD | 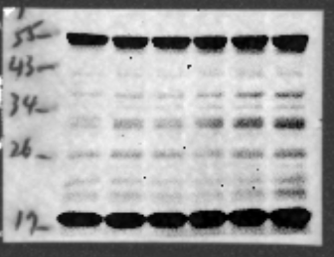 | 31 |
|  | 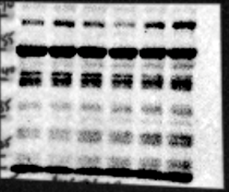 |  |
| actin | 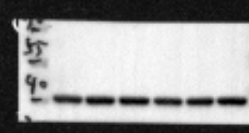 | 42 |
|  | 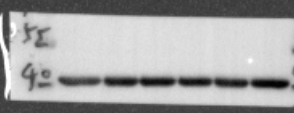 |  |

**S4 Fig. Treatment of sucralose induced the activation of ER stress in hEPCs**

| SCL (mM) | 0 0.02 0.1 0.2 1 2 | M.W. (KDa) |
| --- | --- | --- |
| p-JNK | 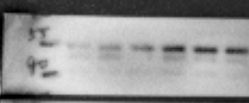 | 54 |
|  | 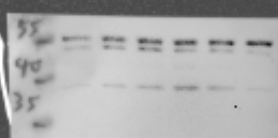 |  |
| p-PERK | 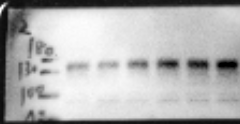 | 150 |
|  | 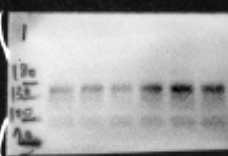 |  |
| p-eIF2 | 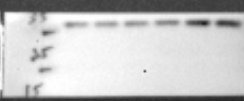 | 36 |
|  | 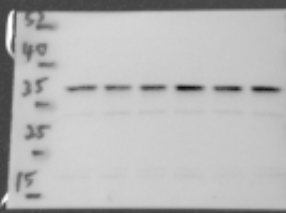 |  |
| Actin | 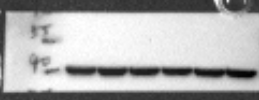 | 42 |
|  | **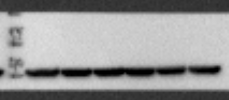** |  |
| CHOP | 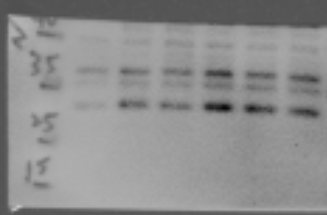 | 27 |
|  | **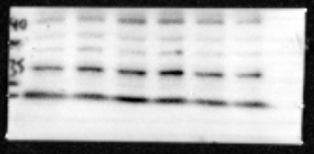** |  |
| Lamin A | 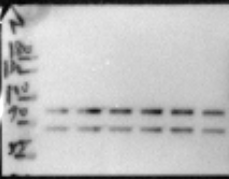 | 70 |
|  | **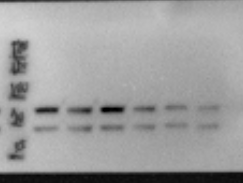** |  |

p-IRE1


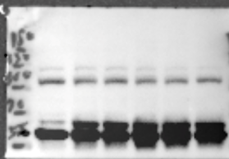
 107


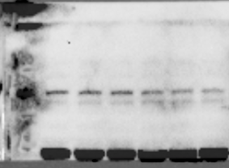


IRE


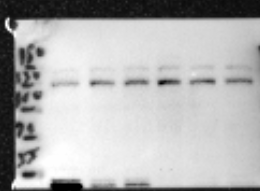


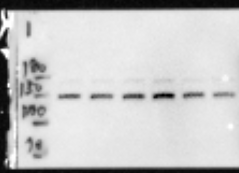
 107

c-ATF6


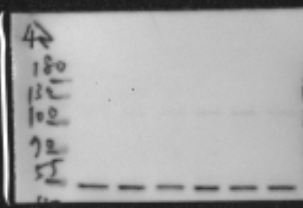
 54


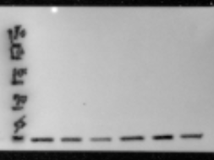


PERK


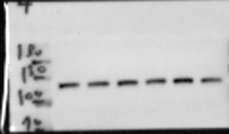
 125

**
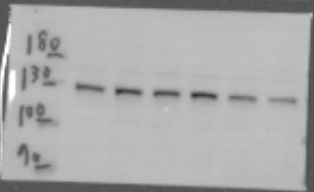
**

eIF2


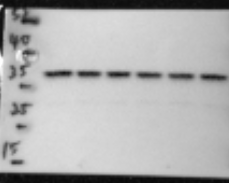
 36


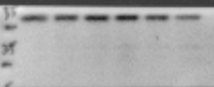


**S5 Fig. Inhibition of ER stress restored cell survival in sucralose-treated hEPCs**

|  |  | M.W. (KDa) |  |
| --- | --- | --- | --- |
| Bax | **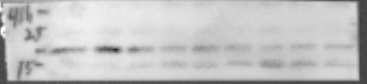** | 20 | |
|  | 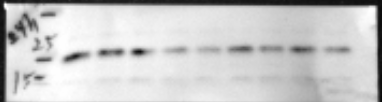 |  | |
| Bcl-2 | **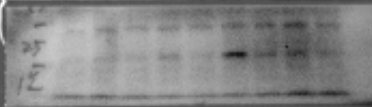** | 23 | |
|  | 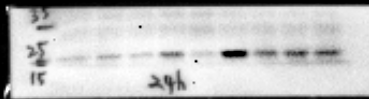 |  | |
| c-caspase3 | 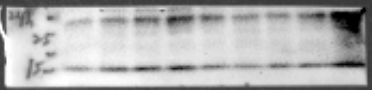 | 17 | |
|  | 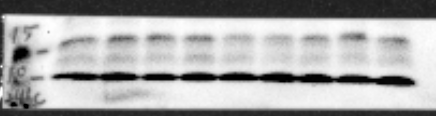 |  | |
| actin | 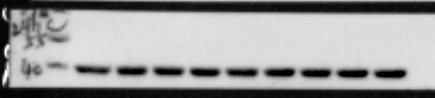 | 42 | |
|  | 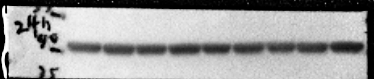 |  | |
| c-PARP | **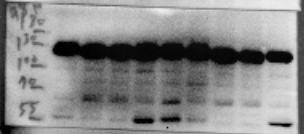** | 89 | |
|  | 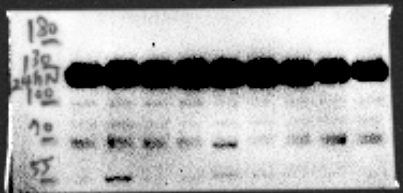 |  | |
| lamin A | 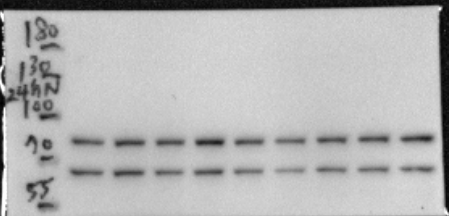 | 70 | |
|  | **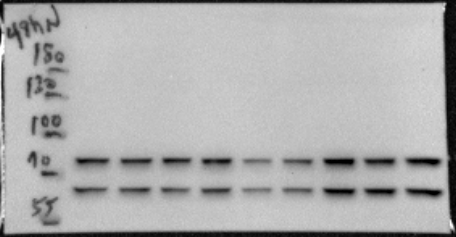** |  | |

| SCL (mM) | 0 0.02 0.1 0.2 1 2 | M.W. (KDa) |
| --- | --- | --- |
| NLRP3 | 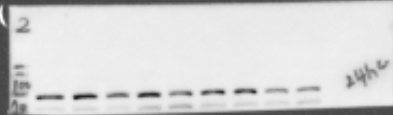 | 110 |
|  | 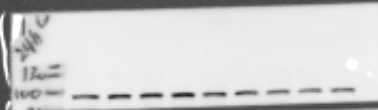 |  |
| c-IL-1β | 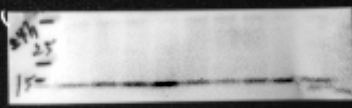 | 17 |
|  | 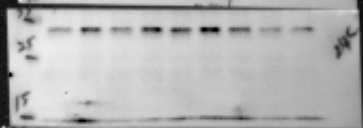 |  |
| c-N-GSDMD | 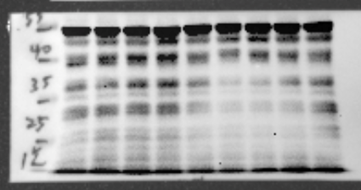 | 31 |
|  | 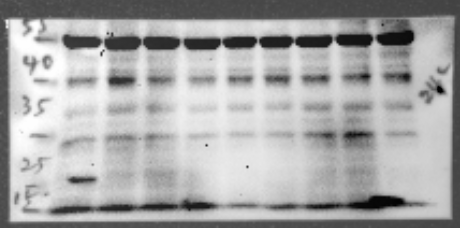 |  |
| actin | 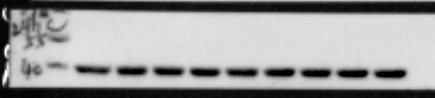 | 42 |
|  | 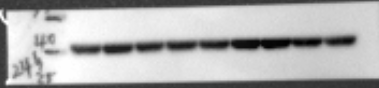 |  |
